# Evaluation of Genetic Associations with Clinical Phenotypes of Kidney Stone Disease

**DOI:** 10.1016/j.euros.2024.07.109

**Published:** 2024-07-24

**Authors:** Ryan S. Hsi, Siwei Zhang, Jefferson L. Triozzi, Adriana M. Hung, Yaomin Xu, Cosmin A. Bejan

**Affiliations:** a Department of Urology, Vanderbilt University Medical Center, Nashville, TN, USA; b Department of Biostatistics, Vanderbilt University Medical Center, Nashville, TN, USA; c Division of Nephrology and Hypertension, Department of Medicine, Vanderbilt University Medical Center, Nashville, TN, USA; d VA Tennessee Valley Healthcare System, Nashville, TN, USA; e Department of Biomedical informatics, Vanderbilt University, Nashville, TN, USA

**Keywords:** Kidney calculi, Nephrolithiasis, Precision phenotyping, Electronic health records, Genome-wide association study

## Abstract

**Background and objective:**

Previous studies have reported a strong genetic contribution to kidney stone risk. This study aims to identify genetic associations of kidney stone disease within a large-scale electronic health record system.

**Methods:**

We performed genome-wide association studies (GWASs) for nephrolithiasis from genotyped samples of 5571 cases and 83 692 controls. This analysis included a primary GWAS focused on nephrolithiasis and subsequent subgroup GWASs stratified by stone composition types. For significant risk variants, we performed association analyses with stone composition and first-time 24-h urine parameters. To assess disease severity, we investigated the associations with age at first stone diagnosis, age at first stone-related procedure, and time between first and second stone-related procedures.

**Key findings and limitations:**

The primary GWAS analysis identified ten significant loci, all located on chromosome 16 within coding regions of the *UMOD* gene. The strongest signal was rs28544423 (odds ratio 1.17, 95% confidence interval 1.11–1.23, *p* = 2.7 × 10^–9^). In subgroup GWASs stratified by six kidney stone composition subtypes, 19 significant loci were identified including two loci in coding regions (brushite; *NXPH1*, rs79970906 and rs4725104). The *UMOD* single nucleotide polymorphism rs28544423 was associated with differences in 24-h excretion of urinary analytes, and the minor allele was positively associated with calcium oxalate dihydrate stone composition *(p* < 0.05). No associations were found between *UMOD* variants and disease severity. Limitations include an omitted variable bias and a misclassification bias.

**Conclusions and clinical implications:**

We replicated germline variants associated with kidney stone disease risk at *UMOD* and reported novel variants associated with stone composition*.* Genetic variants of *UMOD* are associated with differences in 24-h urine parameters and stone composition, but not disease severity.

**Patient summary:**

We identify genetic variants linked to kidney stone disease within an electronic health record (EHR) system. These findings suggest a role for the EHR to enable a precision-medicine approach for stone disease.

## Take Home Message

From a large-scale electronic health record, we replicate kidney stone disease risk loci within *UMOD* (16p12.3). We demonstrate differences in stone composition and 24-h urine parameters consistent with uromodulin function as an inhibitor for calcium-based stone types.

## 1 Introduction

Multiple lines of evidence support a strong genetic contribution to kidney stone risk, including familial studies and twin studies [1], [2], [3]. In addition, prior large-scale genome-wide association studies (GWASs) from the UK, Japan, and Iceland have implicated genetic variants linked to calcium and phosphate regulation, metabolic traits, and oxidative stress [4], [5], [6], [7], [8], [9]. Overall, previous studies have reported that kidney stone risk is approximately 50% attributable to genetic heritability [2], [3].

Despite such a large contribution of genetics to stone risk, clinicians uncommonly utilize genetic information for clinical care for several reasons. First, monogenic causes for kidney stones are uncommon [10], [11]. At present, genetic screening is not performed routinely except for primary hyperoxaluria or cystinuria, and often, these conditions can be inferred indirectly from urine and stone testing. Furthermore, while many genetic studies have elucidated mechanisms for disease risk in population-based epidemiology studies, few have examined these associations with clinical phenotypes such as disease severity in patient clinical context. Additionally, previous research has not explored genetic variants specifically associated with urinary stone composition, a key factor in developing a personalized treatment approach. As such, there is an unmet need to evaluate genetic factors in real-world patient populations, interpret genetic factors linked to patient clinical outcomes, and assess how well these genetic factors may fit into existing treatment algorithms.

Within this context, we investigated the translational potential of using genome-wide association findings. First, we performed a GWAS for kidney stone disease within an electronic health record (EHR) framework. In a subgroup analysis, we performed separate GWASs among subgroups of individuals with kidney stones classified by stone composition. Then, for single nucleotide polymorphisms (SNPs) meeting the criteria for genome-wide significance, we compared differences in 24-h urine values and stone composition by allele status, and evaluated associations with disease severity.

## 2 Patients and methods

### 2.1 Data source and study population

Data were obtained from the Vanderbilt University Medical Center deidentified EHR (Synthetic Derivative [SD]), which is updated bimonthly and contains longitudinal clinical records of 3.2 million records since 1982. The SD is linked to a biobank (BioVU) [12], which has accrued DNA samples since 2007 from unused blood drawn for routine clinical practice scheduled to be discarded. Our study population included individuals within the SD with genotyping data from BioVU (*n* = 90 991). Local institutional review board approval was obtained for this study (IRB# 190480).

### 2.2 Kidney stone cases and controls

We identified cases within the SD using Current Procedural Terminology (CPT) codes and International Classification of Diseases, 9th/10th Revision, Clinical Modification (ICD-9/10-CM) diagnosis codes within the SD up to May 2021. We required a single ICD or CPT code to be classified as a case (Supplementary Table 1). To ensure that these codes reflected kidney stone cases, a manual review of randomly sampled records containing kidney stone–related ICD (*n* = 200) and CPT (*n* = 200) codes showed positive predictive values of 93% and 97%, respectively. Among cases, we identified comorbid conditions prior to the index kidney stone diagnosis based on ICD codes listed in Supplementary Table 2. Controls were identified from noncases who did not have additional diagnosis codes for exclusion (Supplementary Table 1). These exclusion codes included diagnoses of hydronephrosis and lower urinary tract stones.

### 2.3 Genotyping and imputation

Genotyping was performed with the Illumina (San Diego, CA, USA) Expanded Multi-Ethnic Genotyping Array (MEGA^EX^), which includes over 2 million markers, including a comprehensive range of SNPs across exonic, intronic, and intergenic regions, as well as insertions/deletions (indels), and variants relevant to diverse populations and pharmacogenetic studies. The preprocessing protocol and quality control procedures have been described previously [13]. We performed standard quality control procedures to exclude low-quality variants and individuals including SNPs with missingness >2%, individuals with missingness >5%, SNPs with minor allele frequency <1%, and *p* value of Hardy-Weinberg equilibrium test of <1e–6. Then, the data were imputed to the 1000 Genomes Project Phase 3 reference panel [14] for haplotype estimation and imputation. We converted dosage data to hard genotype calls and excluded variants with uncertainty >0.1 or INFO <0.95, resulting in 2 225 361 variants after postimputation quality control.

### 2.4 Primary GWAS

**Supplementary Fig. 2:**
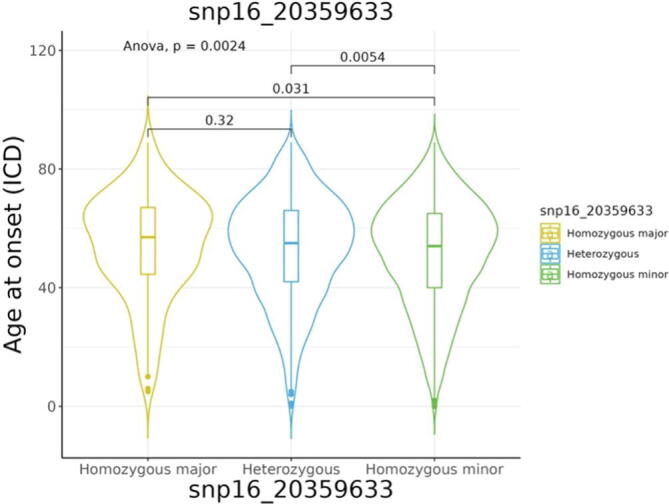
Age at first diagnosis based on SNP rs28544423 allele status. The median age at first stone diagnosis was 54.2 years, 53.1 years, and 51.5 years for homozygous major, heterozygous, and homozygous minor alleles. After accounting for cohort effects, the final analysis showed no significant association (p=0.35).

We performed a GWAS to identify SNPs associated with kidney stone disease. Logistic regression with an additive genetic model was used, adjusting for age at diagnosis, race (White, Black, and other), sex, and ten principal components to control for population stratification (Supplementary Fig. 2) [15], [16]. An additive model assessed the linear increase in risk for each copy of the minor allele. A *p* value of <5 × 10^–8^ was used for genome-wide significance. We additionally performed two sensitivity analyses with more stringent exclusion criteria using additional conditions: (1) at least 2 yr of EHR follow-up data between the timestamp of the first and last records, and (2) nonmissing demographic information (sex, race, and ethnicity). Local linkage disequilibrium (LD) and recombination patterns were accessed using LocusZoom [17]. To isolate the independent signals, we performed a conditional analysis in PLINK by adding the lead SNP to the covariates and rerunning the association test. We utilized ANNOVAR for genomic annotation to provide insight for functional implications [18].

### 2.5 Subgroup GWASs by stone composition

For genotyped individuals, the first stone composition (Beck Analytical Services, Greenwood, IN, USA) from the kidney or ureter was identified. Based on the classifications described previously [19], we performed separate GWASs for majority calcium oxalate (monohydrate and/or dihydrate), majority calcium oxalate monohydrate, majority calcium oxalate dihydrate, majority hydroxyapatite, any uric acid, any brushite, any carbonate apatite, and any struvite. Controls were individuals identified in the main analysis. The GWAS analyses were then performed as described for the main GWAS study.

### 2.6 Comparison of stone composition and 24-h urine

For the significant risk alleles identified in the primary GWAS, individuals were assigned to three groups based on allele status. Stone composition and first-time 24-h urine samples with creatinine/kilogram values within reference ranges (Litholink, Itasca, IL, USA) were identified among those with a kidney stone history. Descriptive statistics and comparisons were performed among the allele groups. Logistic regression models were performed for each stone composition category under dominant, recessive model, and additive genetic models. For each of the 24-h urine parameters, a univariate analysis was performed with an analysis of variance (ANOVA), and *p* values of <0.05 were considered significant.

### 2.7 Disease severity analysis

We assessed the effect of SNP allele status on disease severity for three outcomes: (1) age at first kidney stone diagnosis, (2) age at first surgical procedure, and (3) time to second surgery from first surgical procedure. The first two outcomes were evaluated with ANOVA. To account for the cohort effect associated with the variation among different cohorts at the same age [20], the SNPs with significant results from the Kruskal-Wallis test were evaluated in a linear mixed effect model with sex, race, and ethnicity as fixed effects, and birth cohort (every 10 yr) as a random effect. Finally, the third outcome was assessed using a time to event analysis with Cox proportional hazards regression model adjusting for sex, race, and ethnicity. The log-rank test was used to compare Kaplan-Meier survival curves and determine whether there are statistically significant differences in survival times between the SNP allele groups.

To account for the potential lack of power for the disease severity analyses, we performed equivalence tests for each of the three outcomes. Individuals were split into two groups, with one group having at least one minor risk allele and another group without any minor risk allele. The effect size was converted by the difference in the outcome between the two groups given the sample sizes and a pooled standard deviation.

### 2.8 Software

For the GWAS quality control and association analysis, PLINK 1.9 [21] was used. For the GWAS imputation procedure, IMPUTE2 [22] and SHAPEIT [23] were used. All the other statistical analyses were implemented in R version 4.1.0 (R Foundation for Statistical Computing, Vienna, Austria).

## 3 Results

Among 89 533 genotyped individuals, the analysis included 5571 (6.2%) with kidney stone disease and 83 692 (93.8%) controls. Descriptive statistics for the kidney stone cohort are shown in Supplementary Table 3. The mean age at diagnosis was 52.0 yr, 51.3% were males, and 86.4% were White and 10.5% Black individuals. The most common comorbid conditions were hypertension (52.1%), obesity (24.5%), diabetes type 2 (23.4%), and cardiovascular disease (23.0%).

**Fig. 1:**
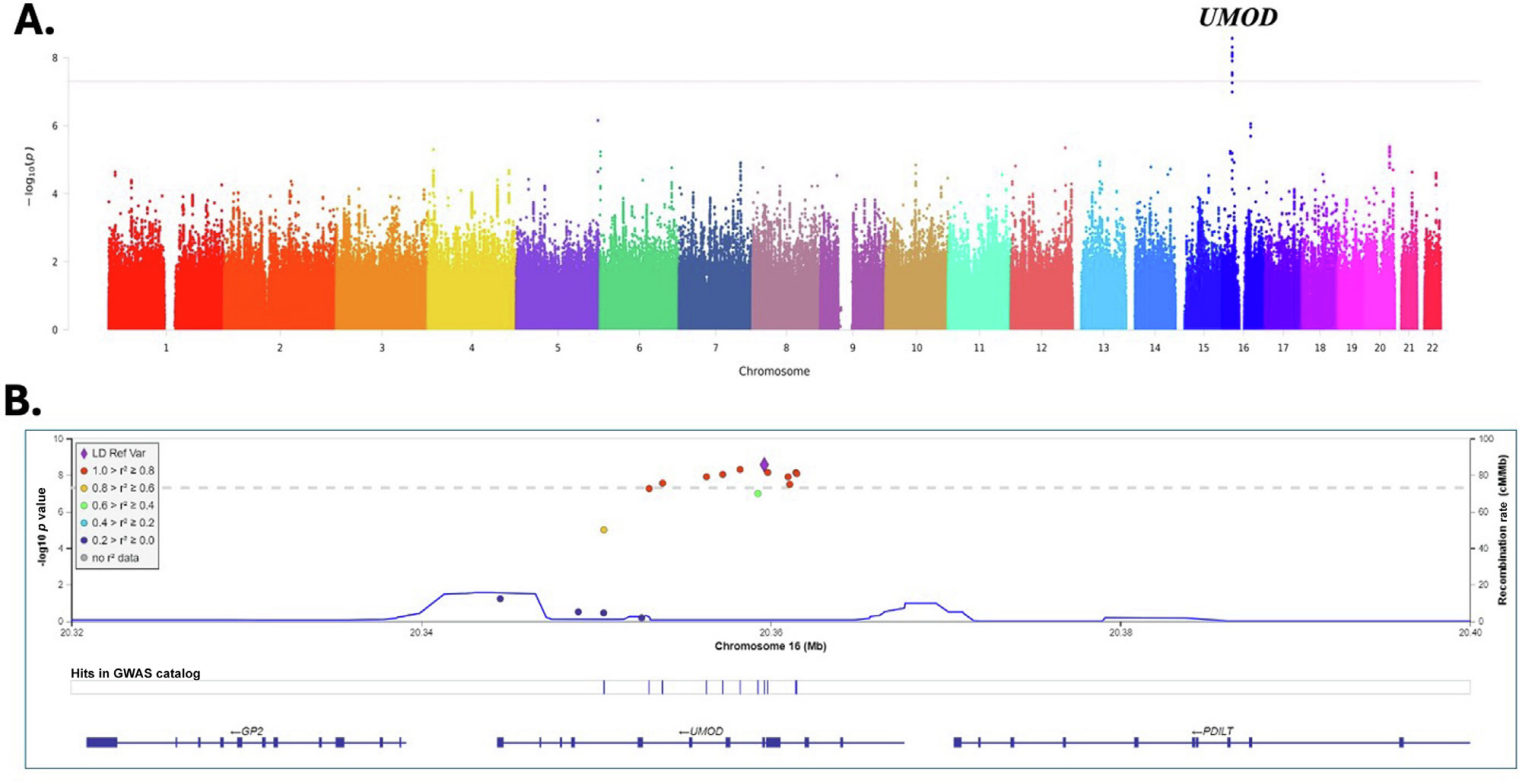
(A) Manhattan plot for the GWAS for kidney stone disease. A *p* value of <5 × 10^–8^ was used for genome-wide significance. (B) LocusZoom depicting GWAS data in the context of chromosome 16 in the *UMOD* region. The data points are colored based on their level of linkage disequilibrium with the index SNP rs2854442. GWAS = genome-wide association study; LD = linkage disequilibrium; SNP = single nucleotide polymorphism.

**Fig. 2:**
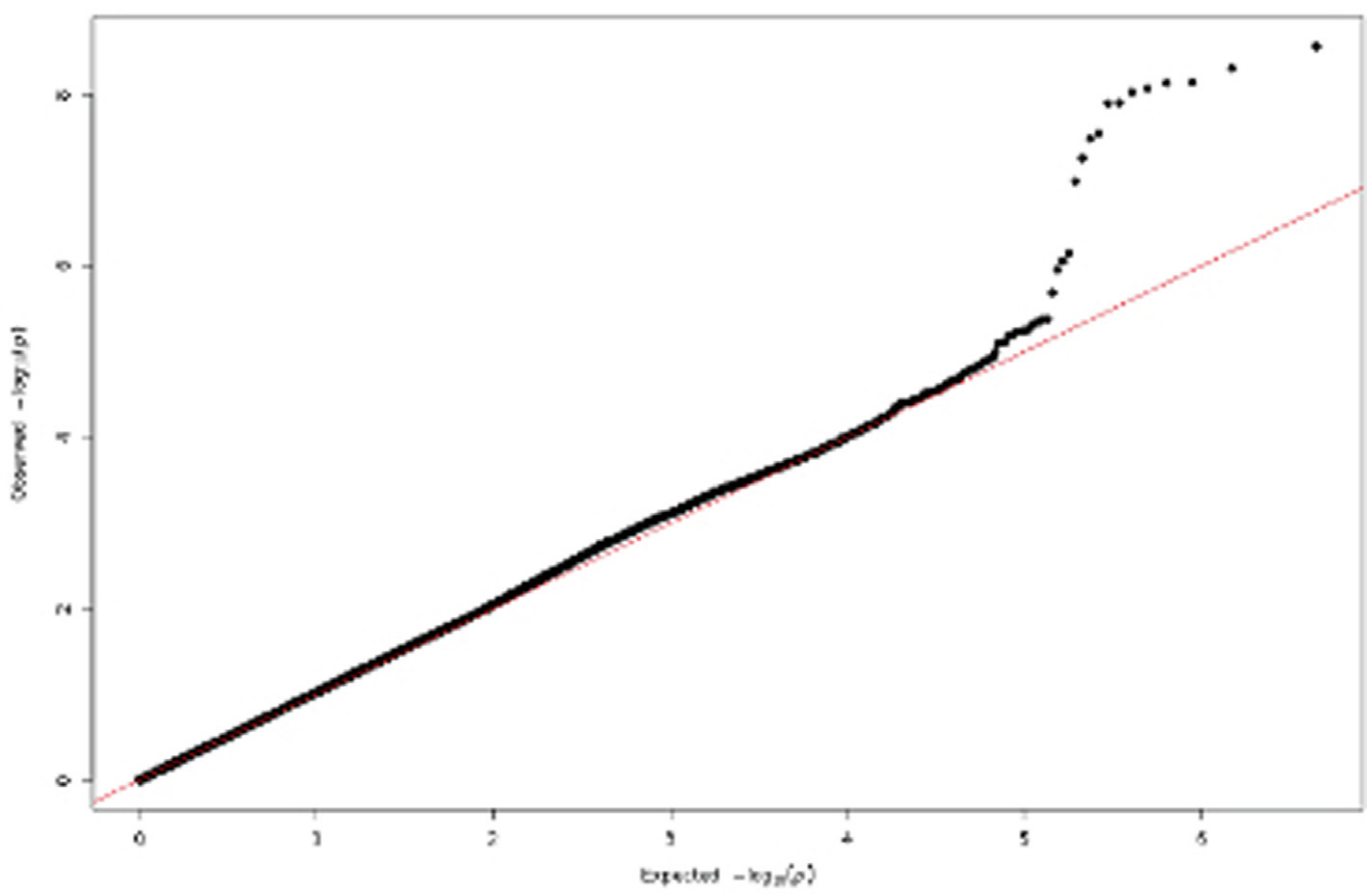
Quantile-quantile plot comparing the observed versus the expected probability distribution. The outliers indicate a subset of variants showing an association with kidney stone disease.

The GWAS results showed ten distinct association signals meeting the genome-wide significance, all located in the *UMOD* gene region on chromosome 16 between 20 344 373 and 20 364 037 bp (GRCh37; Table 1 and Fig. 1A). SNP rs28544423 had the strongest signal, with minor allele positively associated with a higher risk (odds ratio 1.17, 95% confidence interval 1.11–1.23). The conditional analysis indicated that the SNP rs28544423 represents an independent signal, with the associations of all other SNPs vanishing upon adjustment for this specific SNP. There were two additional variants, rs9928936 and rs28640218, within the region of *UMOD* that approached but did not reach the significance threshold. As shown in the LocusZoom and quantile-quantile plots based on the *UMOD* gene region (16p12.3) depicted in Fig. 1, Fig. 2, the associated region and the significant SNPs are in strong LD with the index SNP (r^2^ ≥ 0.8). For the two sensitivity analyses restricting case criteria to the requirement of 2 yr of EHR follow-up data and availability of complete demographic information, the findings were unchanged.

**Table 1:** Genetic variants of kidney stone disease meeting genome-wide significance

Chr	SNP	Minor allele	MAF	OR	95% CI	*p* value	Gene
16	rs28544423	T	0.16	1.17	(1.11, 1.23)	2.7e-09	*UMOD*
16	rs9928003	C	0.16	1.17	(1.11, 1.23)	5.0e-09	*UMOD*
16	rs13335818	T	0.17	1.16	(1.1, 1.22)	7.2e-09	*UMOD*
16	rs34882080	G	0.17	1.16	(1.1, 1.22)	7.3e-09	*UMOD*
16	rs35650857	T	0.17	1.16	(1.1, 1.22)	8.4e-09	*UMOD*
16	rs12934320	T	0.17	1.16	(1.1, 1.22)	9.3e-09	*UMOD*
16	rs34356953	T	0.17	1.16	(1.1, 1.22)	1.2e-08	*UMOD*
16	rs71149135	GCCACATTC	0.16	1.16	(1.1, 1.22)	1.3e-08	*UMOD*
16	rs60136849	C	0.17	1.15	(1.1, 1.21)	2.8e-08	*UMOD*
16	rs111285796	TTCTGCCAGA	0.16	1.16	(1.1, 1.22)	3.3e-08	*UMOD*

Chr = chromosome; CI, confidence interval; MAF = minor allele frequency; OR = odds ratio; SNP = single nucleotide polymorphism.

In the GWASs by kidney stone composition, we identified 19 loci meeting the threshold for genome-wide significance (Table 2). We identified a significant association for brushite stone composition with rs79970906 and rs4725104 in the gene region of *NXPH1* from chromosome 7. For struvite stone composition, our study identified a significant association with SNP rs55726672 on chromosome 8 in a genomic region related to epigenetic function (noncoding RNA LINC01288). This signal was near the significant locus rs186944649 for carbonate apatite on chromosome 8 in an intergenic position between DUSP26 and LINC01288.

**Table 2:** Genetic variants meeting genome-wide significance by kidney stone composition

GWAS	Chr	SNP	Minor allele	OR	95% CI	*p* value	Gene
Majority calcium oxalate dihydrate							
	4	rs56193428	A	4.78	(2.85, 7.99)	2.7e-09	*MIR4455*; *HELT*
	4	rs72706967	T	4.44	(2.66, 7.41)	1.26e-08	*MIR4455*; *HELT*
Majority calcium phosphate (hydroxyapatite)							
	11	rs369841339	A	3.39	(2.23, 5.17)	1.18e-08	*OVCH2*; *OR5P2*
Any uric acid							
	1	rs34398946	C	5.64	(3.08, 10.32)	2.02e-08	*LINC01031*; *LINC01724*
	1	rs35337461	T	5.64	(3.08, 10.32)	2.02e-08	*LINC01031*; *LINC01724*
	1	rs12748379	A	5.60	(3.06, 10.25)	2.29e-08	*LINC01031*; *LINC01724*
	1	rs71642955	G	5.53	(3.02, 10.13)	2.92e-08	*LINC01031*; *LINC01724*
	1	rs35647468	C	5.53	(3.02, 10.12)	2.98e-08	*LINC01031*; *LINC01724*
Any brushite							
	7	rs79970906	G	8.72	(4.35, 17.49)	1.08e-09	*NXPH1*
	7	rs4725104	T	7.72	(3.82, 15.61)	1.24e-08	*NXPH1*
	11	rs148417243	A	46.21	(12.15, 175.84)	1.88e-08	*FAM86C2P*; *UNC93B1*
	11	rs144507654	T	45.26	(11.85, 172.96)	2.49e-08	*FAM86C2P*; *UNC93B1*
	11	rs141950436	G	44.87	(11.78, 170.99)	2.50e-08	*FAM86C2P*; *UNC93B1*
	11	rs72880913	G	10.15	(4.48, 22.99)	2.79e-08	*MIR8054*; *LUZP2*
	11	rs12274909	T	39.43	(10.69, 145.37)	3.40e-08	*FAM86C2P*; *UNC93B1*
	11	rs12273415	A	39.42	(10.68, 145.43)	3.46e-08	*FAM86C2P*; *UNC93B1*
Any carbonate apatite							
	8	rs186944649	A	8.29	(4.00, 17.16)	1.23e-08	*DUSP26*; *LINC01288*
Any struvite							
	8	JHU_8.34686048	T	7.45	(3.85, 14.41)	2.54e-09	*LINC01288*
	3	rs143825102	T	9.32	(4.21, 20.64)	3.66e-08	*OXTR*; *RAD18*

Chr = chromosome; CI = confidence interval; GWAS = genome-wide association study; OR = odds ratio; SNP = single nucleotide polymorphism.

When classifying individuals by allele status for SNP rs28544423, 24-h urine values among 586 individuals differed significantly for 24-h urine calcium, uric acid, phosphorus, and sulfate (each *p* < 0.05; Table 3). Among 1743 individuals with stone composition data, there was a positive association with SNP rs28544423 allele status and majority calcium oxalate dihydrate stone type under either an additive model or a dominant model (*p* < 0.05).

**Table 3:** SNP rs28544423 allele status with 24-h urine and stone composition

24-h urine parameter, mean (SD)	No allele (*N* = 376)	1 allele (*N* = 182)	2 alleles (*N* = 28)	*p* value
Volume (l)	1.85 (0.88)	1.88 (0.88)	1.77 (0.77)	0.784
Calcium (mg)	183 (122)	209 (135)	197 (157)	**0.024**
Oxalate (mg)	37.8 (18.2)	38.4 (18.4)	34.1 (14.8)	0.709
Citrate (mg)	522 (364)	554 (389)	435 (269)	0.345
pH	6.15 (0.56)	6.08 (0.54)	6.24 (0.49)	0.157
Uric acid (g)	0.573 (0.244)	0.618 (0.253)	0.559 (0.252)	**0.044**
Sodium (mEq)	166 (81)	174 (82)	155 (75)	0.258
Potassium (mEq)	56 (26)	57 (28)	55 (29)	0.584
Magnesium (mg)	94 (45)	98 (51)	89 (47)	0.351
Phosphorus (g)	0.876 (0.391)	0.953 (0.401)	0.866 (0.350)	**0.031**
Ammonium (mEq)	33 (18)	34 (17)	35 (27)	0.460
Chloride (mEq)	160 (77)	165 (78)	147 (70)	0.487
Sulfate (mEq)	34 (18)	38 (20)	34 (19)	**0.023**
Creatinine (mg/kg)	13.6 (2.8)	14.1 (2.8)	12.7 (2.8)	0.092
SSCaOx	6.6 (3.9)	7.1 (4.01)	6.3 (3.3)	0.184
SSCaP	1.3 (1.2)	1.3 (1.1)	1.6 (1.3)	0.607
SSUA	0.82 (0.87)	0.96 (0.92)	0.69 (0.78)	0.085

Stone composition (%)	No allele (*N* = 1109)	1 allele (*N* = 551)	2 alleles (*N* = 83)	*p* value *

Majority Calcium Oxalate Monohydrate	31.9	32.3	34.9	0.262
Majority Calcium Oxalate Dihydrate	5.8	8.0	8.4	**0.034**
Majority Calcium Phosphate	13.0	12.3	10.8	0.836
Majority Uric Acid	3.5	2.7	2.4	0.422

* indicates dominant model *p* values are shown. SD = standard deviation; SNP = single nucleotide polymorphism; SSCaOx = supersaturation of calcium oxalate; SSCaP = supersaturation of calcium phosphate; SSUA = supersaturation of uric acid. Bolded *p* value indicates statistical significance at *p*<0.05.

For the disease severity analysis, we considered rs28544423 as the representative SNP. The median age at first stone diagnosis was higher among those with homozygous major (54.2 yr) than among those with heterozygous (53.1 yr, *p* = 0.005) and homozygous (51.5 yr, *p* = 0.03) minor alleles (Supplementary Fig. 2). However, after accounting for cohort effects, the final analysis showed no significant association (*p* = 0.35; Supplementary Table 4). Equivalence testing showed sufficient power to detect a minimum difference of 1 mo.

**Supplementary Fig. 3:**
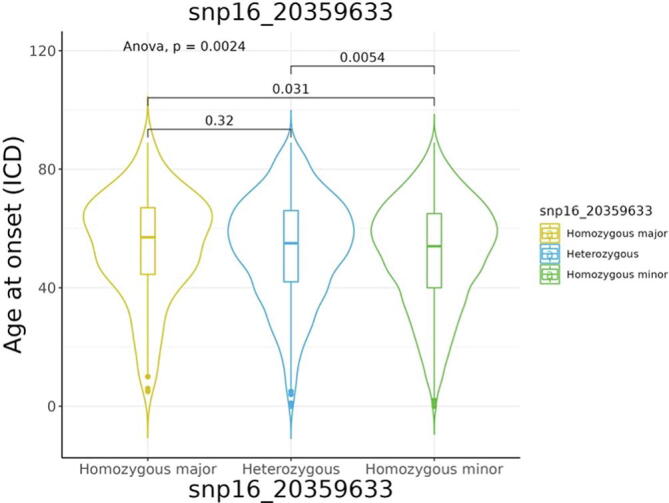
Age at first stone-related surgery based on SNP rs28544423 allele status. After accounting for cohort effects, the final analysis showed no significant association by allele status.

**Supplementary Fig. 4:**
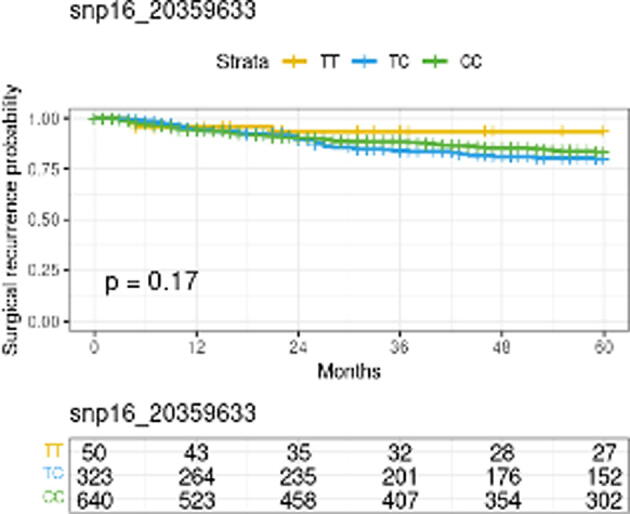
Kaplan-Meier curve showing freedom from second stone-related surgery after first surgery by SNP rs28544423 allele status. CC and TT are homozygous major and minor, respectively. No statistically significant differences were identified comparing time to 2^nd^ surgery at 5 years by allele status.

Among those receiving any kidney stone surgery (*n* = 1106), no differences were observed comparing the age at first surgery or time to second surgery by 5 yr of follow-up (Supplementary Fig. 3 and 4, and Supplementary Table 5). Equivalence testing showed sufficient power to defect a minimum of 1 mo for the age at first surgical procedure outcome and a minimum of 2 mo for the first to second surgery outcome.

## 4 Discussion

We identified several important findings. First, our results provide an independent replication of kidney stone disease risk loci within *UMOD* (16p12.3). *UMOD* encodes uromodulin—also known as Tamm-Horsfall protein, the most abundant protein in urine and an inhibitor of calcium crystallization [24]. We additionally demonstrate differences in stone composition and 24-h urine parameters consistent with uromodulin function as an inhibitor for calcium-based stone types. However, no relationships with kidney stone severity were identified with *UMOD* risk alleles despite adequate statistical power for the outcomes evaluated.

Consistent with other GWASs in British, Icelandic, and Japanese populations [7], [8], [9], *UMOD* genetic variants were associated with a modestly increased risk (17% increased odds) of kidney stone disease in our US-based cohort. The most significant *UMOD* variant we identified is in strong linkage (r^2^ > 0.8) with common variants in the 5′ promoter of the *UMOD* gene that are known to affect urinary uromodulin levels, supporting a causal role [24]. Additionally, uromodulin urinary levels are associated with variants in *PDILT*, which flanks *UMOD* on chromosome 16 [25]. That this *UMOD* variant was also associated with calcium oxalate dihydrate stones, which have been linked to urinary calcium excretion [26], provides additional supportive evidence. In addition to their role in urinary stone risk, *UMOD* variants have been implicated in urinary acidification, renal function, and the development of chronic kidney disease [27], [28], [29]. However, our finding of the lack of association between *UMOD* risk alleles and kidney stone severity suggests that while uromodulin influences kidney stone risk, it may not influence subsequent meaningful clinical outcomes.

In subgroup GWAS analyses on six stone composition subtypes, we implicated 19 novel genome-wide significant loci. Among these, two were located within coding regions, specifically associated with brushite stone disease and the *NXPH1* gene. *NXPH1* codes for Neurexophilin 1, a secreted protein implicated in irritable bowel syndrome [30] and several neurological conditions [31]. Notably, NXPH1 mediates hematopoiesis, immune responses, and osteoblast activity in the bone marrow [32], which could be a plausible pathway to explain the common finding of hypercalciuria among brushite stone formers. The other loci associated with stone composition subtypes were identified predominantly in intergenic regions, and one locus, rs55726672, was found in a noncoding RNA region. These findings suggest potential regulatory roles for these SNPs, and highlight the challenges and complexity of translating these SNPs into clear disease pathways.

There are several limitations to this study. EHR-based datasets are susceptible to an omitted variable bias and a misclassification bias. These data do not capture care sought outside of the health system and would not have identified, for example, surgical procedures at another facility. In addition, these data from the Southeastern USA may not be generalizable to other populations.

Notwithstanding these limitations, our study has important clinical implications. Our findings suggest a role for the EHR to enable a precision-medicine approach for stone disease. A major strength of the EHR is the longitudinal data available across a large population with granular phenotyping data, such as stone composition and 24-h urine data. The replication of *UMOD* from previous GWAS findings validates the EHR as a research data source. We additionally demonstrate that the use of *UMOD* as a biomarker needs further evidence to support its clinical utility within the context of disease severity. Put differently, we show that while genetics may influence risk, this risk may not translate into measurable disease severity. Furthermore, we identify novel risk loci for subgroups by kidney stone composition, and the biological pathways associated with these variations need to be evaluated by functional genomics studies.

## 5 Conclusions

In conclusion, in this EHR-based GWAS, we replicated *UMOD* variants associated with kidney stone disease risk, but these variants were not associated with disease severity. Stone composition and 24-h urine studies comparing *UMOD* variants provide evidence supporting its role in urinary calcium crystallization. We identify novel genetic variants associated with specific subgroups of individuals with kidney stones as classified by stone composition; however, the biological pathways relating to risk need further elucidation. These findings suggest that there may be a role for genetic testing linked to EHRs to facilitate precision-medicine approaches for the treatment of kidney stone disease.

  ***Author contributions*:** Ryan S. Hsi had full access to all the data in the study and takes responsibility for the integrity of the data and the accuracy of the data analysis.

  *Study concept and design*: Hsi, Xu, Bejan.

*Acquisition of data*: Zhang, Bejan.

*Analysis and interpretation of data*: Hsi, Triozzi, Hung, Xu, Bejan.

*Drafting of the manuscript*: Hsi.

*Critical revision of the manuscript for important intellectual content*: Zhang, Xu, Bejan, Triozzi, Hung.

*Statistical analysis*: Zhang, Xu.

*Obtaining funding*: Hsi, Bejan.

*Administrative, technical, or material support*: Hsi.

*Supervision*: Hung.

*Other*: None.

  ***Financial disclosures:*** Ryan S. Hsi certifies that all conflicts of interest, including specific financial interests and relationships and affiliations relevant to the subject matter or materials discussed in the manuscript (eg, employment/affiliation, grants or funding, consultancies, honoraria, stock ownership or options, expert testimony, royalties, or patents filed, received, or pending), are the following: None.

  ***Funding/Support and role of the sponsor*:** This work was supported by National Institutes of Health Grant R21DK127075 and CTSA award no. UL1 TR002243 from NCATS/NIH. Its contents are solely the responsibility of the authors and do not necessarily represent official views of the National Center for Advancing Translational Sciences or the National Institutes of Health.

## Figures and Tables

**Supplementary Fig. 1:**
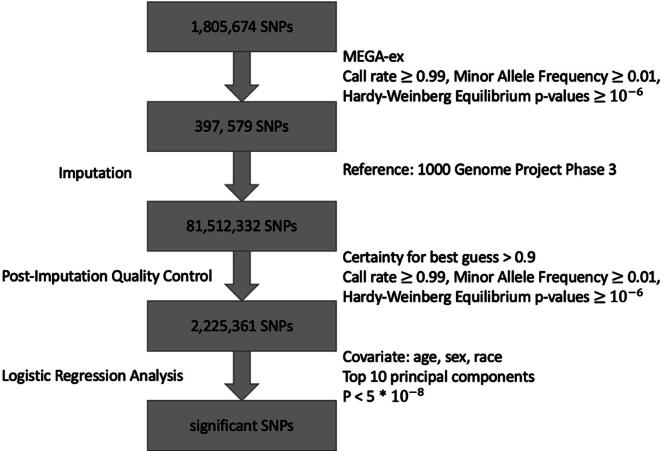
Flow chart of GWAS.
