# Supplementary material for: Evaluation of Genetic Associations with Clinical Phenotypes of Kidney Stone Disease
Source: Eur Urol Open Sci. 2024 Jul 24;67:38–44. doi: 10.1016/j.euros.2024.07.109 (PMC11327546; doi:10.1016/j.euros.2024.07.109)
Supplement: Supplementary Table 1 [file mmc1.docx]

**Supplementary Table 1**. ICD and CPT codes for kidney stone disease

|  | ICD 9 | | ICD 10 |
| --- | --- | --- | --- |
| Diagnosis Codes for inclusion | 592, 592.0, 592.1, 592.9 | | N20, N20.0, N20.1, N20.2, N20.9 |
| CPT Procedure Codes for inclusion | 50080, 50081, 50590, 52320, 52352, 52353, 52356 | | |
| Diagnosis Codes for exclusion | 591, 594, 594.1 594.2, 594.8. 594.9, 788.0, V13.01 | N13.1, N13.2, N13.3, N13.30, N13.39, N21, N21.0, N21.8, N21.9, N22, N23, Z87.442 | |
