# Supplementary material for: Evaluation of Genetic Associations with Clinical Phenotypes of Kidney Stone Disease
Source: Eur Urol Open Sci. 2024 Jul 24;67:38–44. doi: 10.1016/j.euros.2024.07.109 (PMC11327546; doi:10.1016/j.euros.2024.07.109)
Supplement: Supplementary Table 2 [file mmc2.docx]

**Supplementary Table 2**. Diagnosis and procedure codes for kidney stone cases and comorbidities.

**ICD 9**

**Cardiovascular disease**

410 Acute myocardial infarction

410.0 Acute myocardial infarction, of anterolateral wall

410.00 Acute myocardial infarction of anterolateral wall, episode of care unspecified

410.01 Acute myocardial infarction of anterolateral wall, initial episode of care

410.02 Acute myocardial infarction of anterolateral wall, subsequent episode of care

410.1 Acute myocardial infarction, of other anterior wall

410.10 Acute myocardial infarction of other anterior wall, episode of care unspecified

410.11 Acute myocardial infarction of other anterior wall, initial episode of care

410.12 Acute myocardial infarction of other anterior wall, subsequent episode of care

410.2 Acute myocardial infarction, of inferolateral wall

410.20 Acute myocardial infarction of inferolateral wall, episode of care unspecified

410.21 Acute myocardial infarction of inferolateral wall, initial episode of care

410.22 Acute myocardial infarction of inferolateral wall, subsequent episode of care

410.3 Acute myocardial infarction, of inferoposterior wall

410.30 Acute myocardial infarction of inferoposterior wall, episode of care unspecified

410.31 Acute myocardial infarction of inferoposterior wall, initial episode of care

410.32 Acute myocardial infarction of inferoposterior wall, subsequent episode of care

410.4 Acute myocardial infarction, of other inferior wall

410.40 Acute myocardial infarction of other inferior wall, episode of care unspecified

410.41 Acute myocardial infarction of other inferior wall, initial episode of care

410.42 Acute myocardial infarction of other inferior wall, subsequent episode of care

410.5 Acute myocardial infarction, of other lateral wall

410.50 Acute myocardial infarction of other lateral wall, episode of care unspecified

410.51 Acute myocardial infarction of other lateral wall, initial episode of care

410.52 Acute myocardial infarction of other lateral wall, subsequent episode of care

410.6 Acute myocardial infarction, true posterior wall infarction

410.60 True posterior wall infarction, episode of care unspecified

410.61 True posterior wall infarction, initial episode of care

410.62 True posterior wall infarction, subsequent episode of care

410.7 Acute myocardial infarction, subendocardial infarction

410.70 Subendocardial infarction, episode of care unspecified

410.71 Subendocardial infarction, initial episode of care

410.72 Subendocardial infarction, subsequent episode of care

410.8 Acute myocardial infarction, of other specified sites

410.80 Acute myocardial infarction of other specified sites, episode of care unspecified

410.81 Acute myocardial infarction of other specified sites, initial episode of care

410.82 Acute myocardial infarction of other specified sites, subsequent episode of care

410.9 Acute myocardial infarction, unspecified site

410.90 Acute myocardial infarction of unspecified site, episode of care unspecified

410.91 Acute myocardial infarction of unspecified site, initial episode of care

410.92 Acute myocardial infarction of unspecified site, subsequent episode of care

414 Other forms of chronic ischemic heart disease

414.0 Coronary atherosclerosis

414.00 Coronary atherosclerosis of unspecified type of vessel, native or graft

414.01 Coronary atherosclerosis of native coronary artery

414.02 Coronary atherosclerosis of autologous vein bypass graft

414.03 Coronary atherosclerosis of nonautologous biological bypass graft

414.04 Coronary atherosclerosis of artery bypass graft

414.05 Coronary atherosclerosis of unspecified bypass graft

414.06 Coronary atherosclerosis of native coronary artery of transplanted heart

414.07 Coronary atherosclerosis of bypass graft (artery) (vein) of transplanted heart

434 Occlusion of cerebral arteries

434.0 Cerebral thrombosis

434.00 Cerebral thrombosis without mention of cerebral infarction

434.01 Cerebral thrombosis with cerebral infarction

434.1 Cerebral embolism

434.10 Cerebral embolism without mention of cerebral infarction

434.11 Cerebral embolism with cerebral infarction

434.9 Cerebral artery occlusion, unspecified

434.90 Cerebral artery occlusion, unspecified without mention of cerebral infarction

434.91 Cerebral artery occlusion, unspecified with cerebral infarction

**Diabetes Type 2**

250 Diabetes mellitus

250.0 Diabetes mellitus without mention of complication

250.00 Diabetes mellitus without mention of complication, type II or unspecified type, not stated as uncontrolled

250.01 Diabetes mellitus without mention of complication, type I [juvenile type], not stated as uncontrolled

250.02 Diabetes mellitus without mention of complication, type II or unspecified type, uncontrolled

250.03 Diabetes mellitus without mention of complication, type I [juvenile type], uncontrolled

250.1 Diabetes with ketoacidosis

250.10 Diabetes with ketoacidosis, type II or unspecified type, not stated as uncontrolled

250.11 Diabetes with ketoacidosis, type I [juvenile type], not stated as uncontrolled

250.12 Diabetes with ketoacidosis, type II or unspecified type, uncontrolled

250.13 Diabetes with ketoacidosis, type I [juvenile type], uncontrolled

250.2 Diabetes mellitus with hyperosmolarity

250.20 Diabetes with hyperosmolarity, type II or unspecified type, not stated as uncontrolled

250.21 Diabetes with hyperosmolarity, type I [juvenile type], not stated as uncontrolled

250.22 Diabetes with hyperosmolarity, type II or unspecified type, uncontrolled

250.23 Diabetes with hyperosmolarity, type I [juvenile type], uncontrolled

250.3 Diabetes with other coma

250.30 Diabetes with other coma, type II or unspecified type, not stated as uncontrolled

250.31 Diabetes with other coma, type I [juvenile type], not stated as uncontrolled

250.32 Diabetes with other coma, type II or unspecified type, uncontrolled

250.33 Diabetes with other coma, type I [juvenile type], uncontrolled

250.4 Diabetes with renal manifestations

250.40 Diabetes with renal manifestations, type II or unspecified type, not stated as uncontrolled

250.41 Diabetes with renal manifestations, type I [juvenile type], not stated as uncontrolled

250.42 Diabetes with renal manifestations, type II or unspecified type, uncontrolled

250.43 Diabetes with renal manifestations, type I [juvenile type], uncontrolled

250.5 Diabetes with ophthalmic manifestations

250.50 Diabetes with ophthalmic manifestations, type II or unspecified type, not stated as uncontrolled

250.51 Diabetes with ophthalmic manifestations, type I [juvenile type], not stated as uncontrolled

250.52 Diabetes with ophthalmic manifestations, type II or unspecified type, uncontrolled

250.53 Diabetes with ophthalmic manifestations, type I [juvenile type], uncontrolled

250.6 Diabetes with neurological manifestations

250.60 Diabetes with neurological manifestations, type II or unspecified type, not stated as uncontrolled

250.61 Diabetes with neurological manifestations, type I [juvenile type], not stated as uncontrolled

250.62 Diabetes with neurological manifestations, type II or unspecified type, uncontrolled

250.63 Diabetes with neurological manifestations, type I [juvenile type], uncontrolled

250.7 Diabetes with peripheral circulatory disorders

250.70 Diabetes with peripheral circulatory disorders, type II or unspecified type, not stated as uncontrolled

250.71 Diabetes with peripheral circulatory disorders, type I [juvenile type], not stated as uncontrolled

250.72 Diabetes with peripheral circulatory disorders, type II or unspecified type, uncontrolled

250.73 Diabetes with peripheral circulatory disorders, type I [juvenile type], uncontrolled

250.8 Diabetes with other specified manifestations

250.80 Diabetes with other specified manifestations, type II or unspecified type, not stated as uncontrolled

250.81 Diabetes with other specified manifestations, type I [juvenile type], not stated as uncontrolled

250.82 Diabetes with other specified manifestations, type II or unspecified type, uncontrolled

250.83 Diabetes with other specified manifestations, type I [juvenile type], uncontrolled

250.9 Diabetes with unspecified complication

250.90 Diabetes with unspecified complication, type II or unspecified type, not stated as uncontrolled

250.91 Diabetes with unspecified complication, type I [juvenile type], not stated as uncontrolled

250.92 Diabetes with unspecified complication, type II or unspecified type, uncontrolled Gout

**Hypertension**

401 Essential hypertension

401.0 Malignant essential hypertension

401.1 Benign essential hypertension

401.9 Unspecified essential hypertension

**Inflammatory Bowel Disease/Malabsorption Syndromes**

555 Regional enteritis

555.0 Regional enteritis of small intestine

555.1 Regional enteritis of large intestine

555.2 Regional enteritis of small intestine with large intestine

555.9 Regional enteritis of unspecified site

556 Ulcerative colitis

556.0 Ulcerative (chronic) enterocolitis

556.1 Ulcerative (chronic) ileocolitis

556.2 Ulcerative (chronic) proctitis

556.3 Ulcerative (chronic) proctosigmoiditis

556.4 Pseudopolyposis of colon

556.5 Left-sided ulcerative (chronic) colitis

556.6 Universal ulcerative (chronic) colitis

556.8 Other ulcerative colitis

556.9 Ulcerative colitis, unspecified

579 Intestinal malabsorption

579.0 Celiac disease

579.1 Tropical sprue

579.2 Blind loop syndrome

579.3 Other and unspecified postsurgical nonabsorption

579.4 Pancreatic steatorrhea

579.8 Other specified intestinal malabsorption

579.9 Unspecified intestinal malabsorption

V45.3 Intestinal bypass or anastomosis status

V45.86 Bariatric surgery status

**Obesity**

278 Overweight, obesity and other hyperalimentation

278.0 Overweight and obesity

278.00 Obesity, unspecified

278.01 Morbid obesity

V85.3 Body Mass Index between 30-39, adult

V85.30 Body Mass Index 30.0-30.9, adult

V85.31 Body Mass Index 31.0-31.9, adult

V85.32 Body Mass Index 32.0-32.9, adult

V85.33 Body Mass Index 33.0-33.9, adult

V85.34 Body Mass Index 34.0-34.9, adult

V85.35 Body Mass Index 35.0-35.9, adult

V85.36 Body Mass Index 36.0-36.9, adult

V85.37 Body Mass Index 37.0-37.9, adult

V85.38 Body Mass Index 38.0-38.9, adult

V85.39 Body Mass Index 39.0-39.9, adult

V85.4 Body Mass Index 40 and over, adult

V85.41 Body Mass Index 40.0-44.9, adult

V85.42 Body Mass Index 45.0-49.9, adult

V85.43 Body Mass Index 50.0-59.9, adult

V85.44 Body Mass Index 60.0-69.9, adult

V85.45 Body Mass Index 70 and over, adult

**ICD 10**

**Cardiovascular disease**

I21 Acute myocardial infarction

I21.0 ST elevation (STEMI) myocardial infarction of anterior wall

I21.01 ST elevation (STEMI) myocardial infarction involving left main coronary artery

I21.02 ST elevation (STEMI) myocardial infarction involving left anterior descending coronary artery

I21.09 ST elevation (STEMI) myocardial infarction involving other coronary artery of anterior wall

I21.1 ST elevation (STEMI) myocardial infarction of inferior wall

I21.11 ST elevation (STEMI) myocardial infarction involving right coronary artery

I21.19 ST elevation (STEMI) myocardial infarction involving other coronary artery of inferior wall

I21.2 ST elevation (STEMI) myocardial infarction of other sites

I21.21 ST elevation (STEMI) myocardial infarction involving left circumflex coronary artery

I21.29 ST elevation (STEMI) myocardial infarction involving other sites

I21.3 ST elevation (STEMI) myocardial infarction of unspecified site

I21.4 Non-ST elevation (NSTEMI) myocardial infarction

I21.9 Acute myocardial infarction, unspecified

I21.A Other type of myocardial infarction

I21.A1 Myocardial infarction type 2

I21.A9 Other myocardial infarction type

I25 Chronic ischemic heart disease

I25.1 Atherosclerotic heart disease of native coronary artery

I25.10 Atherosclerotic heart disease of native coronary artery without angina pectoris

I25.11 Atherosclerotic heart disease of native coronary artery with angina pectoris

I25.110 Atherosclerotic heart disease of native coronary artery with unstable angina pectoris

I25.111 Atherosclerotic heart disease of native coronary artery with angina pectoris with documented spasm

I25.118 Atherosclerotic heart disease of native coronary artery with other forms of angina pectoris

I25.119 Atherosclerotic heart disease of native coronary artery with unspecified angina pectoris

I25.2 Old myocardial infarction

I25.3 Aneurysm of heart

I25.4 Coronary artery aneurysm and dissection

I25.41 Coronary artery aneurysm

I25.42 Coronary artery dissection

I25.5 Ischemic cardiomyopathy

I25.6 Silent myocardial ischemia

I25.7 Atherosclerosis of coronary artery bypass graft(s) and coronary artery of transplanted heart with angina pectoris

I25.70 Atherosclerosis of coronary artery bypass graft(s), unspecified, with angina pectoris

I25.700 Atherosclerosis of coronary artery bypass graft(s), unspecified, with unstable angina pectoris

I25.701 Atherosclerosis of coronary artery bypass graft(s), unspecified, with angina pectoris with documented spasm

I25.708 Atherosclerosis of coronary artery bypass graft(s), unspecified, with other forms of angina pectoris

I25.709 Atherosclerosis of coronary artery bypass graft(s), unspecified, with unspecified angina pectoris

I25.71 Atherosclerosis of autologous vein coronary artery bypass graft(s) with angina pectoris

I25.710 Atherosclerosis of autologous vein coronary artery bypass graft(s) with unstable angina pectoris

I25.711 Atherosclerosis of autologous vein coronary artery bypass graft(s) with angina pectoris with documented spasm

I25.718 Atherosclerosis of autologous vein coronary artery bypass graft(s) with other forms of angina pectoris

I25.719 Atherosclerosis of autologous vein coronary artery bypass graft(s) with unspecified angina pectoris

I25.72 Atherosclerosis of autologous artery coronary artery bypass graft(s) with angina pectoris

I25.720 Atherosclerosis of autologous artery coronary artery bypass graft(s) with unstable angina pectoris

I25.721 Atherosclerosis of autologous artery coronary artery bypass graft(s) with angina pectoris with documented spasm

I25.728 Atherosclerosis of autologous artery coronary artery bypass graft(s) with other forms of angina pectoris

I25.729 Atherosclerosis of autologous artery coronary artery bypass graft(s) with unspecified angina pectoris

I25.73 Atherosclerosis of nonautologous biological coronary artery bypass graft(s) with angina pectoris

I25.730 Atherosclerosis of nonautologous biological coronary artery bypass graft(s) with unstable angina pectoris

I25.731 Atherosclerosis of nonautologous biological coronary artery bypass graft(s) with angina pectoris with documented spasm

I25.738 Atherosclerosis of nonautologous biological coronary artery bypass graft(s) with other forms of angina pectoris

I25.739 Atherosclerosis of nonautologous biological coronary artery bypass graft(s) with unspecified angina pectoris

I25.75 Atherosclerosis of native coronary artery of transplanted heart with angina pectoris

I25.750 Atherosclerosis of native coronary artery of transplanted heart with unstable angina

I25.751 Atherosclerosis of native coronary artery of transplanted heart with angina pectoris with documented spasm

I25.758 Atherosclerosis of native coronary artery of transplanted heart with other forms of angina pectoris

I25.759 Atherosclerosis of native coronary artery of transplanted heart with unspecified angina pectoris

I25.76 Atherosclerosis of bypass graft of coronary artery of transplanted heart with angina pectoris

I25.760 Atherosclerosis of bypass graft of coronary artery of transplanted heart with unstable angina

I25.761 Atherosclerosis of bypass graft of coronary artery of transplanted heart with angina pectoris with documented spasm

I25.768 Atherosclerosis of bypass graft of coronary artery of transplanted heart with other forms of angina pectoris

I25.769 Atherosclerosis of bypass graft of coronary artery of transplanted heart with unspecified angina pectoris

I25.79 Atherosclerosis of other coronary artery bypass graft(s) with angina pectoris

I25.790 Atherosclerosis of other coronary artery bypass graft(s) with unstable angina pectoris

I25.791 Atherosclerosis of other coronary artery bypass graft(s) with angina pectoris with documented spasm

I25.798 Atherosclerosis of other coronary artery bypass graft(s) with other forms of angina pectoris

I25.799 Atherosclerosis of other coronary artery bypass graft(s) with unspecified angina pectoris

I25.8 Other forms of chronic ischemic heart disease

I25.81 Atherosclerosis of other coronary vessels without angina pectoris

I25.810 Atherosclerosis of coronary artery bypass graft(s) without angina pectoris

I25.811 Atherosclerosis of native coronary artery of transplanted heart without angina pectoris

I25.812 Atherosclerosis of bypass graft of coronary artery of transplanted heart without angina pectoris

I25.82 Chronic total occlusion of coronary artery

I25.83 Coronary atherosclerosis due to lipid rich plaque

I25.84 Coronary atherosclerosis due to calcified coronary lesion

I25.89 Other forms of chronic ischemic heart disease

I25.9 Chronic ischemic heart disease, unspecified

I63 Cerebral infarction

I63.0 Cerebral infarction due to thrombosis of precerebral arteries

I63.00 Cerebral infarction due to thrombosis of unspecified precerebral artery

I63.01 Cerebral infarction due to thrombosis of vertebral artery

I63.011 Cerebral infarction due to thrombosis of right vertebral artery

I63.012 Cerebral infarction due to thrombosis of left vertebral artery

I63.013 Cerebral infarction due to thrombosis of bilateral vertebral arteries

I63.019 Cerebral infarction due to thrombosis of unspecified vertebral artery

I63.02 Cerebral infarction due to thrombosis of basilar artery

I63.03 Cerebral infarction due to thrombosis of carotid artery

I63.031 Cerebral infarction due to thrombosis of right carotid artery

I63.032 Cerebral infarction due to thrombosis of left carotid artery

I63.033 Cerebral infarction due to thrombosis of bilateral carotid arteries

I63.039 Cerebral infarction due to thrombosis of unspecified carotid artery

I63.09 Cerebral infarction due to thrombosis of other precerebral artery

I63.1 Cerebral infarction due to embolism of precerebral arteries

I63.10 Cerebral infarction due to embolism of unspecified precerebral artery

I63.11 Cerebral infarction due to embolism of vertebral artery

I63.111 Cerebral infarction due to embolism of right vertebral artery

I63.112 Cerebral infarction due to embolism of left vertebral artery

I63.113 Cerebral infarction due to embolism of bilateral vertebral arteries

I63.119 Cerebral infarction due to embolism of unspecified vertebral artery

I63.12 Cerebral infarction due to embolism of basilar artery

I63.13 Cerebral infarction due to embolism of carotid artery

I63.131 Cerebral infarction due to embolism of right carotid artery

I63.132 Cerebral infarction due to embolism of left carotid artery

I63.133 Cerebral infarction due to embolism of bilateral carotid arteries

I63.139 Cerebral infarction due to embolism of unspecified carotid artery

I63.19 Cerebral infarction due to embolism of other precerebral artery

I63.2 Cerebral infarction due to unspecified occlusion or stenosis of precerebral arteries

I63.20 Cerebral infarction due to unspecified occlusion or stenosis of unspecified precerebral arteries

I63.21 Cerebral infarction due to unspecified occlusion or stenosis of vertebral arteries

I63.211 Cerebral infarction due to unspecified occlusion or stenosis of right vertebral artery

I63.212 Cerebral infarction due to unspecified occlusion or stenosis of left vertebral artery

I63.213 Cerebral infarction due to unspecified occlusion or stenosis of bilateral vertebral arteries

I63.219 Cerebral infarction due to unspecified occlusion or stenosis of unspecified vertebral artery

I63.22 Cerebral infarction due to unspecified occlusion or stenosis of basilar artery

I63.23 Cerebral infarction due to unspecified occlusion or stenosis of carotid arteries

I63.231 Cerebral infarction due to unspecified occlusion or stenosis of right carotid arteries

I63.232 Cerebral infarction due to unspecified occlusion or stenosis of left carotid arteries

I63.233 Cerebral infarction due to unspecified occlusion or stenosis of bilateral carotid arteries

I63.239 Cerebral infarction due to unspecified occlusion or stenosis of unspecified carotid artery

I63.29 Cerebral infarction due to unspecified occlusion or stenosis of other precerebral arteries

I63.3 Cerebral infarction due to thrombosis of cerebral arteries

I63.30 Cerebral infarction due to thrombosis of unspecified cerebral artery

I63.31 Cerebral infarction due to thrombosis of middle cerebral artery

I63.311 Cerebral infarction due to thrombosis of right middle cerebral artery

I63.312 Cerebral infarction due to thrombosis of left middle cerebral artery

I63.313 Cerebral infarction due to thrombosis of bilateral middle cerebral arteries

I63.319 Cerebral infarction due to thrombosis of unspecified middle cerebral artery

I63.32 Cerebral infarction due to thrombosis of anterior cerebral artery

I63.321 Cerebral infarction due to thrombosis of right anterior cerebral artery

I63.322 Cerebral infarction due to thrombosis of left anterior cerebral artery

I63.323 Cerebral infarction due to thrombosis of bilateral anterior cerebral arteries

I63.329 Cerebral infarction due to thrombosis of unspecified anterior cerebral artery

I63.33 Cerebral infarction due to thrombosis of posterior cerebral artery

I63.331 Cerebral infarction due to thrombosis of right posterior cerebral artery

I63.332 Cerebral infarction due to thrombosis of left posterior cerebral artery

I63.333 Cerebral infarction due to thrombosis of bilateral posterior cerebral arteries

I63.339 Cerebral infarction due to thrombosis of unspecified posterior cerebral artery

I63.34 Cerebral infarction due to thrombosis of cerebellar artery

I63.341 Cerebral infarction due to thrombosis of right cerebellar artery

I63.342 Cerebral infarction due to thrombosis of left cerebellar artery

I63.343 Cerebral infarction due to thrombosis of bilateral cerebellar arteries

I63.349 Cerebral infarction due to thrombosis of unspecified cerebellar artery

I63.39 Cerebral infarction due to thrombosis of other cerebral artery

I63.4 Cerebral infarction due to embolism of cerebral arteries

I63.40 Cerebral infarction due to embolism of unspecified cerebral artery

I63.41 Cerebral infarction due to embolism of middle cerebral artery

I63.411 Cerebral infarction due to embolism of right middle cerebral artery

I63.412 Cerebral infarction due to embolism of left middle cerebral artery

I63.413 Cerebral infarction due to embolism of bilateral middle cerebral arteries

I63.419 Cerebral infarction due to embolism of unspecified middle cerebral artery

I63.42 Cerebral infarction due to embolism of anterior cerebral artery

I63.421 Cerebral infarction due to embolism of right anterior cerebral artery

I63.422 Cerebral infarction due to embolism of left anterior cerebral artery

I63.423 Cerebral infarction due to embolism of bilateral anterior cerebral arteries

I63.429 Cerebral infarction due to embolism of unspecified anterior cerebral artery

I63.43 Cerebral infarction due to embolism of posterior cerebral artery

I63.431 Cerebral infarction due to embolism of right posterior cerebral artery

I63.432 Cerebral infarction due to embolism of left posterior cerebral artery

I63.433 Cerebral infarction due to embolism of bilateral posterior cerebral arteries

I63.439 Cerebral infarction due to embolism of unspecified posterior cerebral artery

I63.44 Cerebral infarction due to embolism of cerebellar artery

I63.441 Cerebral infarction due to embolism of right cerebellar artery

I63.442 Cerebral infarction due to embolism of left cerebellar artery

I63.443 Cerebral infarction due to embolism of bilateral cerebellar arteries

I63.449 Cerebral infarction due to embolism of unspecified cerebellar artery

I63.49 Cerebral infarction due to embolism of other cerebral artery

I63.5 Cerebral infarction due to unspecified occlusion or stenosis of cerebral arteries

I63.50 Cerebral infarction due to unspecified occlusion or stenosis of unspecified cerebral artery

I63.51 Cerebral infarction due to unspecified occlusion or stenosis of middle cerebral artery

I63.511 Cerebral infarction due to unspecified occlusion or stenosis of right middle cerebral artery

I63.512 Cerebral infarction due to unspecified occlusion or stenosis of left middle cerebral artery

I63.513 Cerebral infarction due to unspecified occlusion or stenosis of bilateral middle cerebral arteries

I63.519 Cerebral infarction due to unspecified occlusion or stenosis of unspecified middle cerebral artery

I63.52 Cerebral infarction due to unspecified occlusion or stenosis of anterior cerebral artery

I63.521 Cerebral infarction due to unspecified occlusion or stenosis of right anterior cerebral artery

I63.522 Cerebral infarction due to unspecified occlusion or stenosis of left anterior cerebral artery

I63.523 Cerebral infarction due to unspecified occlusion or stenosis of bilateral anterior cerebral arteries

I63.529 Cerebral infarction due to unspecified occlusion or stenosis of unspecified anterior cerebral artery

I63.53 Cerebral infarction due to unspecified occlusion or stenosis of posterior cerebral artery

I63.531 Cerebral infarction due to unspecified occlusion or stenosis of right posterior cerebral artery

I63.532 Cerebral infarction due to unspecified occlusion or stenosis of left posterior cerebral artery

I63.533 Cerebral infarction due to unspecified occlusion or stenosis of bilateral posterior cerebral arteries

I63.539 Cerebral infarction due to unspecified occlusion or stenosis of unspecified posterior cerebral artery

I63.54 Cerebral infarction due to unspecified occlusion or stenosis of cerebellar artery

I63.541 Cerebral infarction due to unspecified occlusion or stenosis of right cerebellar artery

I63.542 Cerebral infarction due to unspecified occlusion or stenosis of left cerebellar artery

I63.543 Cerebral infarction due to unspecified occlusion or stenosis of bilateral cerebellar arteries

I63.549 Cerebral infarction due to unspecified occlusion or stenosis of unspecified cerebellar artery

I63.59 Cerebral infarction due to unspecified occlusion or stenosis of other cerebral artery

I63.6 Cerebral infarction due to cerebral venous thrombosis, nonpyogenic

I63.8 Other cerebral infarction

I63.81 Other cerebral infarction due to occlusion or stenosis of small artery

I63.89 Other cerebral infarction

I63.9 Cerebral infarction, unspecified

**Diabetes Type 2**

E11 Type 2 diabetes mellitus

E11.0 Type 2 diabetes mellitus with hyperosmolarity

E11.00 Type 2 diabetes mellitus with hyperosmolarity without nonketotic hyperglycemic-hyperosmolar coma (NKHHC)

E11.01 Type 2 diabetes mellitus with hyperosmolarity with coma

E11.1 Type 2 diabetes mellitus with ketoacidosis

E11.10 Type 2 diabetes mellitus with ketoacidosis without coma

E11.11 Type 2 diabetes mellitus with ketoacidosis with coma

E11.2 Type 2 diabetes mellitus with kidney complications

E11.21 Type 2 diabetes mellitus with diabetic nephropathy

E11.22 Type 2 diabetes mellitus with diabetic chronic kidney disease

E11.29 Type 2 diabetes mellitus with other diabetic kidney complication

E11.3 Type 2 diabetes mellitus with ophthalmic complications

E11.31 Type 2 diabetes mellitus with unspecified diabetic retinopathy

E11.311 Type 2 diabetes mellitus with unspecified diabetic retinopathy with macular edema

E11.319 Type 2 diabetes mellitus with unspecified diabetic retinopathy without macular edema

E11.32 Type 2 diabetes mellitus with mild nonproliferative diabetic retinopathy

E11.321 Type 2 diabetes mellitus with mild nonproliferative diabetic retinopathy with macular edema

E11.3211 Type 2 diabetes mellitus with mild nonproliferative diabetic retinopathy with macular edema, right eye

E11.3212 Type 2 diabetes mellitus with mild nonproliferative diabetic retinopathy with macular edema, left eye

E11.3213 Type 2 diabetes mellitus with mild nonproliferative diabetic retinopathy with macular edema, bilateral

E11.3219 Type 2 diabetes mellitus with mild nonproliferative diabetic retinopathy with macular edema, unspecified eye

E11.329 Type 2 diabetes mellitus with mild nonproliferative diabetic retinopathy without macular edema

E11.3291 Type 2 diabetes mellitus with mild nonproliferative diabetic retinopathy without macular edema, right eye

E11.3292 Type 2 diabetes mellitus with mild nonproliferative diabetic retinopathy without macular edema, left eye

E11.3293 Type 2 diabetes mellitus with mild nonproliferative diabetic retinopathy without macular edema, bilateral

E11.3299 Type 2 diabetes mellitus with mild nonproliferative diabetic retinopathy without macular edema, unspecified eye

E11.33 Type 2 diabetes mellitus with moderate nonproliferative diabetic retinopathy

E11.331 Type 2 diabetes mellitus with moderate nonproliferative diabetic retinopathy with macular edema

E11.3311 Type 2 diabetes mellitus with moderate nonproliferative diabetic retinopathy with macular edema, right eye

E11.3312 Type 2 diabetes mellitus with moderate nonproliferative diabetic retinopathy with macular edema, left eye

E11.3313 Type 2 diabetes mellitus with moderate nonproliferative diabetic retinopathy with macular edema, bilateral

E11.3319 Type 2 diabetes mellitus with moderate nonproliferative diabetic retinopathy with macular edema, unspecified eye

E11.339 Type 2 diabetes mellitus with moderate nonproliferative diabetic retinopathy without macular edema

E11.3391 Type 2 diabetes mellitus with moderate nonproliferative diabetic retinopathy without macular edema, right eye

E11.3392 Type 2 diabetes mellitus with moderate nonproliferative diabetic retinopathy without macular edema, left eye

E11.3393 Type 2 diabetes mellitus with moderate nonproliferative diabetic retinopathy without macular edema, bilateral

E11.3399 Type 2 diabetes mellitus with moderate nonproliferative diabetic retinopathy without macular edema, unspecified eye

E11.34 Type 2 diabetes mellitus with severe nonproliferative diabetic retinopathy

E11.341 Type 2 diabetes mellitus with severe nonproliferative diabetic retinopathy with macular edema

E11.3411 Type 2 diabetes mellitus with severe nonproliferative diabetic retinopathy with macular edema, right eye

E11.3412 Type 2 diabetes mellitus with severe nonproliferative diabetic retinopathy with macular edema, left eye

E11.3413 Type 2 diabetes mellitus with severe nonproliferative diabetic retinopathy with macular edema, bilateral

E11.3419 Type 2 diabetes mellitus with severe nonproliferative diabetic retinopathy with macular edema, unspecified eye

E11.349 Type 2 diabetes mellitus with severe nonproliferative diabetic retinopathy without macular edema

E11.3491 Type 2 diabetes mellitus with severe nonproliferative diabetic retinopathy without macular edema, right eye

E11.3492 Type 2 diabetes mellitus with severe nonproliferative diabetic retinopathy without macular edema, left eye

E11.3493 Type 2 diabetes mellitus with severe nonproliferative diabetic retinopathy without macular edema, bilateral

E11.3499 Type 2 diabetes mellitus with severe nonproliferative diabetic retinopathy without macular edema, unspecified eye

E11.35 Type 2 diabetes mellitus with proliferative diabetic retinopathy

E11.351 Type 2 diabetes mellitus with proliferative diabetic retinopathy with macular edema

E11.3511 Type 2 diabetes mellitus with proliferative diabetic retinopathy with macular edema, right eye

E11.3512 Type 2 diabetes mellitus with proliferative diabetic retinopathy with macular edema, left eye

E11.3513 Type 2 diabetes mellitus with proliferative diabetic retinopathy with macular edema, bilateral

E11.3519 Type 2 diabetes mellitus with proliferative diabetic retinopathy with macular edema, unspecified eye

E11.352 Type 2 diabetes mellitus with proliferative diabetic retinopathy with traction retinal detachment involving the macula

E11.3521 Type 2 diabetes mellitus with proliferative diabetic retinopathy with traction retinal detachment involving the macula, right eye

E11.3522 Type 2 diabetes mellitus with proliferative diabetic retinopathy with traction retinal detachment involving the macula, left eye

E11.3523 Type 2 diabetes mellitus with proliferative diabetic retinopathy with traction retinal detachment involving the macula, bilateral

E11.3529 Type 2 diabetes mellitus with proliferative diabetic retinopathy with traction retinal detachment involving the macula, unspecified eye

E11.353 Type 2 diabetes mellitus with proliferative diabetic retinopathy with traction retinal detachment not involving the macula

E11.3531 Type 2 diabetes mellitus with proliferative diabetic retinopathy with traction retinal detachment not involving the macula, right eye

E11.3532 Type 2 diabetes mellitus with proliferative diabetic retinopathy with traction retinal detachment not involving the macula, left eye

E11.3533 Type 2 diabetes mellitus with proliferative diabetic retinopathy with traction retinal detachment not involving the macula, bilateral

E11.3539 Type 2 diabetes mellitus with proliferative diabetic retinopathy with traction retinal detachment not involving the macula, unspecified eye

E11.354 Type 2 diabetes mellitus with proliferative diabetic retinopathy with combined traction retinal detachment and rhegmatogenous retinal detachment

E11.3541 Type 2 diabetes mellitus with proliferative diabetic retinopathy with combined traction retinal detachment and rhegmatogenous retinal detachment, right eye

E11.3542 Type 2 diabetes mellitus with proliferative diabetic retinopathy with combined traction retinal detachment and rhegmatogenous retinal detachment, left eye

E11.3543 Type 2 diabetes mellitus with proliferative diabetic retinopathy with combined traction retinal detachment and rhegmatogenous retinal detachment, bilateral

E11.3549 Type 2 diabetes mellitus with proliferative diabetic retinopathy with combined traction retinal detachment and rhegmatogenous retinal detachment, unspecified eye

E11.355 Type 2 diabetes mellitus with stable proliferative diabetic retinopathy

E11.3551 Type 2 diabetes mellitus with stable proliferative diabetic retinopathy, right eye

E11.3552 Type 2 diabetes mellitus with stable proliferative diabetic retinopathy, left eye

E11.3553 Type 2 diabetes mellitus with stable proliferative diabetic retinopathy, bilateral

E11.3559 Type 2 diabetes mellitus with stable proliferative diabetic retinopathy, unspecified eye

E11.359 Type 2 diabetes mellitus with proliferative diabetic retinopathy without macular edema

E11.3591 Type 2 diabetes mellitus with proliferative diabetic retinopathy without macular edema, right eye

E11.3592 Type 2 diabetes mellitus with proliferative diabetic retinopathy without macular edema, left eye

E11.3593 Type 2 diabetes mellitus with proliferative diabetic retinopathy without macular edema, bilateral

E11.3599 Type 2 diabetes mellitus with proliferative diabetic retinopathy without macular edema, unspecified eye

E11.36 Type 2 diabetes mellitus with diabetic cataract

E11.37 Type 2 diabetes mellitus with diabetic macular edema, resolved following treatment

E11.37X1 Type 2 diabetes mellitus with diabetic macular edema, resolved following treatment, right eye

E11.37X2 Type 2 diabetes mellitus with diabetic macular edema, resolved following treatment, left eye

E11.37X3 Type 2 diabetes mellitus with diabetic macular edema, resolved following treatment, bilateral

E11.37X9 Type 2 diabetes mellitus with diabetic macular edema, resolved following treatment, unspecified eye

E11.39 Type 2 diabetes mellitus with other diabetic ophthalmic complication

E11.4 Type 2 diabetes mellitus with neurological complications

E11.40 Type 2 diabetes mellitus with diabetic neuropathy, unspecified

E11.41 Type 2 diabetes mellitus with diabetic mononeuropathy

E11.42 Type 2 diabetes mellitus with diabetic polyneuropathy

E11.43 Type 2 diabetes mellitus with diabetic autonomic (poly)neuropathy

E11.44 Type 2 diabetes mellitus with diabetic amyotrophy

E11.49 Type 2 diabetes mellitus with other diabetic neurological complication

E11.5 Type 2 diabetes mellitus with circulatory complications

E11.51 Type 2 diabetes mellitus with diabetic peripheral angiopathy without gangrene

E11.52 Type 2 diabetes mellitus with diabetic peripheral angiopathy with gangrene

E11.59 Type 2 diabetes mellitus with other circulatory complications

E11.6 Type 2 diabetes mellitus with other specified complications

E11.61 Type 2 diabetes mellitus with diabetic arthropathy

E11.610 Type 2 diabetes mellitus with diabetic neuropathic arthropathy

E11.618 Type 2 diabetes mellitus with other diabetic arthropathy

E11.62 Type 2 diabetes mellitus with skin complications

E11.620 Type 2 diabetes mellitus with diabetic dermatitis

E11.621 Type 2 diabetes mellitus with foot ulcer

E11.622 Type 2 diabetes mellitus with other skin ulcer

E11.628 Type 2 diabetes mellitus with other skin complications

E11.63 Type 2 diabetes mellitus with oral complications

E11.630 Type 2 diabetes mellitus with periodontal disease

E11.638 Type 2 diabetes mellitus with other oral complications

E11.64 Type 2 diabetes mellitus with hypoglycemia

E11.640 Type 2 diabetes mellitus with hypoglycemia without coma

E11.641 Type 2 diabetes mellitus with hypoglycemia with coma

E11.649 Type 2 diabetes mellitus with hypoglycemia without coma

E11.65 Type 2 diabetes mellitus with hyperglycemia

E11.69 Type 2 diabetes mellitus with other specified complication

E11.8 Type 2 diabetes mellitus with unspecified complications

E11.9 Type 2 diabetes mellitus without complications

E13 Other specified diabetes mellitus

E13.0 Other specified diabetes mellitus with hyperosmolarity

E13.00 Other specified diabetes mellitus with hyperosmolarity without nonketotic hyperglycemic-hyperosmolar coma (NKHHC)

E13.01 Other specified diabetes mellitus with hyperosmolarity with coma

E13.1 Other specified diabetes mellitus with ketoacidosis

E13.10 Other specified diabetes mellitus with ketoacidosis without coma

E13.11 Other specified diabetes mellitus with ketoacidosis with coma

E13.2 Other specified diabetes mellitus with kidney complications

E13.21 Other specified diabetes mellitus with diabetic nephropathy

E13.22 Other specified diabetes mellitus with diabetic chronic kidney disease

E13.29 Other specified diabetes mellitus with other diabetic kidney complication

E13.3 Other specified diabetes mellitus with ophthalmic complications

E13.31 Other specified diabetes mellitus with unspecified diabetic retinopathy

E13.311 Other specified diabetes mellitus with unspecified diabetic retinopathy with macular edema

E13.319 Other specified diabetes mellitus with unspecified diabetic retinopathy without macular edema

E13.32 Other specified diabetes mellitus with mild nonproliferative diabetic retinopathy

E13.321 Other specified diabetes mellitus with mild nonproliferative diabetic retinopathy with macular edema

E13.3211 Other specified diabetes mellitus with mild nonproliferative diabetic retinopathy with macular edema, right eye

E13.3212 Other specified diabetes mellitus with mild nonproliferative diabetic retinopathy with macular edema, left eye

E13.3213 Other specified diabetes mellitus with mild nonproliferative diabetic retinopathy with macular edema, bilateral

E13.3219 Other specified diabetes mellitus with mild nonproliferative diabetic retinopathy with macular edema, unspecified eye

E13.329 Other specified diabetes mellitus with mild nonproliferative diabetic retinopathy without macular edema

E13.3291 Other specified diabetes mellitus with mild nonproliferative diabetic retinopathy without macular edema, right eye

E13.3292 Other specified diabetes mellitus with mild nonproliferative diabetic retinopathy without macular edema, left eye

E13.3293 Other specified diabetes mellitus with mild nonproliferative diabetic retinopathy without macular edema, bilateral

E13.3299 Other specified diabetes mellitus with mild nonproliferative diabetic retinopathy without macular edema, unspecified eye

E13.33 Other specified diabetes mellitus with moderate nonproliferative diabetic retinopathy

E13.331 Other specified diabetes mellitus with moderate nonproliferative diabetic retinopathy with macular edema

E13.3311 Other specified diabetes mellitus with moderate nonproliferative diabetic retinopathy with macular edema, right eye

E13.3312 Other specified diabetes mellitus with moderate nonproliferative diabetic retinopathy with macular edema, left eye

E13.3313 Other specified diabetes mellitus with moderate nonproliferative diabetic retinopathy with macular edema, bilateral

E13.3319 Other specified diabetes mellitus with moderate nonproliferative diabetic retinopathy with macular edema, unspecified eye

E13.339 Other specified diabetes mellitus with moderate nonproliferative diabetic retinopathy without macular edema

E13.3391 Other specified diabetes mellitus with moderate nonproliferative diabetic retinopathy without macular edema, right eye

E13.3392 Other specified diabetes mellitus with moderate nonproliferative diabetic retinopathy without macular edema, left eye

E13.3393 Other specified diabetes mellitus with moderate nonproliferative diabetic retinopathy without macular edema, bilateral

E13.3399 Other specified diabetes mellitus with moderate nonproliferative diabetic retinopathy without macular edema, unspecified eye

E13.34 Other specified diabetes mellitus with severe nonproliferative diabetic retinopathy

E13.341 Other specified diabetes mellitus with severe nonproliferative diabetic retinopathy with macular edema

E13.3411 Other specified diabetes mellitus with severe nonproliferative diabetic retinopathy with macular edema, right eye

E13.3412 Other specified diabetes mellitus with severe nonproliferative diabetic retinopathy with macular edema, left eye

E13.3413 Other specified diabetes mellitus with severe nonproliferative diabetic retinopathy with macular edema, bilateral

E13.3419 Other specified diabetes mellitus with severe nonproliferative diabetic retinopathy with macular edema, unspecified eye

E13.349 Other specified diabetes mellitus with severe nonproliferative diabetic retinopathy without macular edema

E13.3491 Other specified diabetes mellitus with severe nonproliferative diabetic retinopathy without macular edema, right eye

E13.3492 Other specified diabetes mellitus with severe nonproliferative diabetic retinopathy without macular edema, left eye

E13.3493 Other specified diabetes mellitus with severe nonproliferative diabetic retinopathy without macular edema, bilateral

E13.3499 Other specified diabetes mellitus with severe nonproliferative diabetic retinopathy without macular edema, unspecified eye

E13.35 Other specified diabetes mellitus with proliferative diabetic retinopathy

E13.351 Other specified diabetes mellitus with proliferative diabetic retinopathy with macular edema

E13.3511 Other specified diabetes mellitus with proliferative diabetic retinopathy with macular edema, right eye

E13.3512 Other specified diabetes mellitus with proliferative diabetic retinopathy with macular edema, left eye

E13.3513 Other specified diabetes mellitus with proliferative diabetic retinopathy with macular edema, bilateral

E13.3519 Other specified diabetes mellitus with proliferative diabetic retinopathy with macular edema, unspecified eye

E13.352 Other specified diabetes mellitus with proliferative diabetic retinopathy with traction retinal detachment involving the macula

E13.3521 Other specified diabetes mellitus with proliferative diabetic retinopathy with traction retinal detachment involving the macula, right eye

E13.3522 Other specified diabetes mellitus with proliferative diabetic retinopathy with traction retinal detachment involving the macula, left eye

E13.3523 Other specified diabetes mellitus with proliferative diabetic retinopathy with traction retinal detachment involving the macula, bilateral

E13.3529 Other specified diabetes mellitus with proliferative diabetic retinopathy with traction retinal detachment involving the macula, unspecified eye

E13.353 Other specified diabetes mellitus with proliferative diabetic retinopathy with traction retinal detachment not involving the macula

E13.3531 Other specified diabetes mellitus with proliferative diabetic retinopathy with traction retinal detachment not involving the macula, right eye

E13.3532 Other specified diabetes mellitus with proliferative diabetic retinopathy with traction retinal detachment not involving the macula, left eye

E13.3533 Other specified diabetes mellitus with proliferative diabetic retinopathy with traction retinal detachment not involving the macula, bilateral

E13.3539 Other specified diabetes mellitus with proliferative diabetic retinopathy with traction retinal detachment not involving the macula, unspecified eye

E13.354 Other specified diabetes mellitus with proliferative diabetic retinopathy with combined traction retinal detachment and rhegmatogenous retinal detachment

E13.3541 Other specified diabetes mellitus with proliferative diabetic retinopathy with combined traction retinal detachment and rhegmatogenous retinal detachment, right eye

E13.3542 Other specified diabetes mellitus with proliferative diabetic retinopathy with combined traction retinal detachment and rhegmatogenous retinal detachment, left eye

E13.3543 Other specified diabetes mellitus with proliferative diabetic retinopathy with combined traction retinal detachment and rhegmatogenous retinal detachment, bilateral

E13.3549 Other specified diabetes mellitus with proliferative diabetic retinopathy with combined traction retinal detachment and rhegmatogenous retinal detachment, unspecified eye

E13.355 Other specified diabetes mellitus with stable proliferative diabetic retinopathy

E13.3551 Other specified diabetes mellitus with stable proliferative diabetic retinopathy, right eye

E13.3552 Other specified diabetes mellitus with stable proliferative diabetic retinopathy, left eye

E13.3553 Other specified diabetes mellitus with stable proliferative diabetic retinopathy, bilateral

E13.3559 Other specified diabetes mellitus with stable proliferative diabetic retinopathy, unspecified eye

E13.359 Other specified diabetes mellitus with proliferative diabetic retinopathy without macular edema

E13.3591 Other specified diabetes mellitus with proliferative diabetic retinopathy without macular edema, right eye

E13.3592 Other specified diabetes mellitus with proliferative diabetic retinopathy without macular edema, left eye

E13.3593 Other specified diabetes mellitus with proliferative diabetic retinopathy without macular edema, bilateral

E13.3599 Other specified diabetes mellitus with proliferative diabetic retinopathy without macular edema, unspecified eye

E13.36 Other specified diabetes mellitus with diabetic cataract

E13.37 Other specified diabetes mellitus with diabetic macular edema, resolved following treatment

E13.37X1 Other specified diabetes mellitus with diabetic macular edema, resolved following treatment, right eye

E13.37X2 Other specified diabetes mellitus with diabetic macular edema, resolved following treatment, left eye

E13.37X3 Other specified diabetes mellitus with diabetic macular edema, resolved following treatment, bilateral

E13.37X9 Other specified diabetes mellitus with diabetic macular edema, resolved following treatment, unspecified eye

E13.39 Other specified diabetes mellitus with other diabetic ophthalmic complication

E13.4 Other specified diabetes mellitus with neurological complications

E13.40 Other specified diabetes mellitus with diabetic neuropathy, unspecified

E13.41 Other specified diabetes mellitus with diabetic mononeuropathy

E13.42 Other specified diabetes mellitus with diabetic polyneuropathy

E13.43 Other specified diabetes mellitus with diabetic autonomic (poly)neuropathy

E13.44 Other specified diabetes mellitus with diabetic amyotrophy

E13.49 Other specified diabetes mellitus with other diabetic neurological complication

E13.5 Other specified diabetes mellitus with circulatory complications

E13.51 Other specified diabetes mellitus with diabetic peripheral angiopathy without gangrene

E13.52 Other specified diabetes mellitus with diabetic peripheral angiopathy with gangrene

E13.59 Other specified diabetes mellitus with other circulatory complications

E13.6 Other specified diabetes mellitus with other specified complications

E13.61 Other specified diabetes mellitus with diabetic arthropathy

E13.610 Other specified diabetes mellitus with diabetic neuropathic arthropathy

E13.618 Other specified diabetes mellitus with other diabetic arthropathy

E13.62 Other specified diabetes mellitus with skin complications

E13.620 Other specified diabetes mellitus with diabetic dermatitis

E13.621 Other specified diabetes mellitus with foot ulcer

E13.622 Other specified diabetes mellitus with other skin ulcer

E13.628 Other specified diabetes mellitus with other skin complications

E13.63 Other specified diabetes mellitus with oral complications

E13.630 Other specified diabetes mellitus with periodontal disease

E13.638 Other specified diabetes mellitus with other oral complications

E13.64 Other specified diabetes mellitus with hypoglycemia

E13.640 Other specified diabetes mellitus with hypoglycemia without coma

E13.641 Other specified diabetes mellitus with hypoglycemia with coma

E13.649 Other specified diabetes mellitus with hypoglycemia without coma

E13.65 Other specified diabetes mellitus with hyperglycemia

E13.69 Other specified diabetes mellitus with other specified complication

E13.8 Other specified diabetes mellitus with unspecified complications

E13.9 Other specified diabetes mellitus without complications

**Hypertension**

I10 Essential (primary) hypertension

I11 Hypertensive heart disease

I11.0 Hypertensive heart disease with heart failure

I11.9 Hypertensive heart disease without heart failure

I12 Hypertensive chronic kidney disease

I12.0 Hypertensive chronic kidney disease with stage 5 chronic kidney disease or end stage renal disease

I12.9 Hypertensive chronic kidney disease with stage 1 through stage 4 chronic kidney disease, or unspecified chronic kidney disease

I13 Hypertensive heart and chronic kidney disease

I13.0 Hypertensive heart and chronic kidney disease with heart failure and stage 1 through stage 4 chronic kidney disease, or unspecified chronic kidney disease

I13.1 Hypertensive heart and chronic kidney disease without heart failure

I13.10 Hypertensive heart and chronic kidney disease without heart failure, with stage 1 through stage 4 chronic kidney disease, or unspecified chronic kidney disease

I13.11 Hypertensive heart and chronic kidney disease without heart failure, with stage 5 chronic kidney disease, or end stage renal disease

I13.2 Hypertensive heart and chronic kidney disease with heart failure and with stage 5 chronic kidney disease, or end stage renal disease

I15 Secondary hypertension

I15.0 Renovascular hypertension

I15.1 Hypertension secondary to other renal disorders

I15.2 Hypertension secondary to endocrine disorders

I15.8 Other secondary hypertension

I15.9 Secondary hypertension, unspecified

I16 Hypertensive crisis

I16.0 Hypertensive urgency

I16.1 Hypertensive emergency

I16.9 Hypertensive crisis, unspecified

**Inflammatory Bowel Disease/Malabsorption Syndromes**

K50.011 Crohn's disease of small intestine with rectal bleeding

K50.012 Crohn's disease of small intestine with intestinal obstruction

K50.013 Crohn's disease of small intestine with fistula

K50.014 Crohn's disease of small intestine with abscess

K50.018 Crohn's disease of small intestine with other complication

K50.019 Crohn's disease of small intestine with unspecified complications

K50.1 Crohn's disease of large intestine

K50.10 Crohn's disease of large intestine without complications

K50.11 Crohn's disease of large intestine with complications

K50.111 Crohn's disease of large intestine with rectal bleeding

K50.112 Crohn's disease of large intestine with intestinal obstruction

K50.113 Crohn's disease of large intestine with fistula

K50.114 Crohn's disease of large intestine with abscess

K50.118 Crohn's disease of large intestine with other complication

K50.119 Crohn's disease of large intestine with unspecified complications

K50.8 Crohn's disease of both small and large intestine

K50.80 Crohn's disease of both small and large intestine without complications

K50.81 Crohn's disease of both small and large intestine with complications

K50.811 Crohn's disease of both small and large intestine with rectal bleeding

K50.812 Crohn's disease of both small and large intestine with intestinal obstruction

K50.813 Crohn's disease of both small and large intestine with fistula

K50.814 Crohn's disease of both small and large intestine with abscess

K50.818 Crohn's disease of both small and large intestine with other complication

K50.819 Crohn's disease of both small and large intestine with unspecified complications

K50.9 Crohn's disease, unspecified

K50.90 Crohn's disease, unspecified, without complications

K50.91 Crohn's disease, unspecified, with complications

K50.911 Crohn's disease, unspecified, with rectal bleeding

K50.912 Crohn's disease, unspecified, with intestinal obstruction

K50.913 Crohn's disease, unspecified, with fistula

K50.914 Crohn's disease, unspecified, with abscess

K50.918 Crohn's disease, unspecified, with other complication

K50.919 Crohn's disease, unspecified, with unspecified complications

K51 Ulcerative colitis

K51.0 Ulcerative (chronic) pancolitis

K51.00 Ulcerative (chronic) pancolitis without complications

K51.01 Ulcerative (chronic) pancolitis with complications

K51.011 Ulcerative (chronic) pancolitis with rectal bleeding

K51.012 Ulcerative (chronic) pancolitis with intestinal obstruction

K51.013 Ulcerative (chronic) pancolitis with fistula

K51.014 Ulcerative (chronic) pancolitis with abscess

K51.018 Ulcerative (chronic) pancolitis with other complication

K51.019 Ulcerative (chronic) pancolitis with unspecified complications

K51.2 Ulcerative (chronic) proctitis

K51.20 Ulcerative (chronic) proctitis without complications

K51.21 Ulcerative (chronic) proctitis with complications

K51.211 Ulcerative (chronic) proctitis with rectal bleeding

K51.212 Ulcerative (chronic) proctitis with intestinal obstruction

K51.213 Ulcerative (chronic) proctitis with fistula

K51.214 Ulcerative (chronic) proctitis with abscess

K51.218 Ulcerative (chronic) proctitis with other complication

K51.219 Ulcerative (chronic) proctitis with unspecified complications

K51.3 Ulcerative (chronic) rectosigmoiditis

K51.30 Ulcerative (chronic) rectosigmoiditis without complications

K51.31 Ulcerative (chronic) rectosigmoiditis with complications

K51.311 Ulcerative (chronic) rectosigmoiditis with rectal bleeding

K51.312 Ulcerative (chronic) rectosigmoiditis with intestinal obstruction

K51.313 Ulcerative (chronic) rectosigmoiditis with fistula

K51.314 Ulcerative (chronic) rectosigmoiditis with abscess

K51.318 Ulcerative (chronic) rectosigmoiditis with other complication

K51.319 Ulcerative (chronic) rectosigmoiditis with unspecified complications

K51.4 Inflammatory polyps of colon

K51.40 Inflammatory polyps of colon without complications

K51.41 Inflammatory polyps of colon with complications

K51.411 Inflammatory polyps of colon with rectal bleeding

K51.412 Inflammatory polyps of colon with intestinal obstruction

K51.413 Inflammatory polyps of colon with fistula

K51.414 Inflammatory polyps of colon with abscess

K51.418 Inflammatory polyps of colon with other complication

K51.419 Inflammatory polyps of colon with unspecified complications

K51.5 Left sided colitis

K51.50 Left sided colitis without complications

K51.51 Left sided colitis with complications

K51.511 Left sided colitis with rectal bleeding

K51.512 Left sided colitis with intestinal obstruction

K51.513 Left sided colitis with fistula

K51.514 Left sided colitis with abscess

K51.518 Left sided colitis with other complication

K51.519 Left sided colitis with unspecified complications

K51.8 Other ulcerative colitis

K51.80 Other ulcerative colitis without complications

K51.81 Other ulcerative colitis with complications

K51.811 Other ulcerative colitis with rectal bleeding

K51.812 Other ulcerative colitis with intestinal obstruction

K51.813 Other ulcerative colitis with fistula

K51.814 Other ulcerative colitis with abscess

K51.818 Other ulcerative colitis with other complication

K51.819 Other ulcerative colitis with unspecified complications

K51.9 Ulcerative colitis, unspecified

K51.90 Ulcerative colitis, unspecified, without complications

K51.91 Ulcerative colitis, unspecified, with complications

K51.911 Ulcerative colitis, unspecified with rectal bleeding

K51.912 Ulcerative colitis, unspecified with intestinal obstruction

K51.913 Ulcerative colitis, unspecified with fistula

K51.914 Ulcerative colitis, unspecified with abscess

K51.918 Ulcerative colitis, unspecified with other complication

K51.919 Ulcerative colitis, unspecified with unspecified complications

K90.1 Tropical sprue

K90.2 Blind loop syndrome, not elsewhere classified

K90.3 Pancreatic steatorrhea

K90.4 Other malabsorption due to intolerance

K90.41 Non-celiac gluten sensitivity

K90.49 Malabsorption due to intolerance, not elsewhere classified

K90.8 Other intestinal malabsorption

K90.81 Whipple's disease

K90.89 Other intestinal malabsorption

K90.9 Intestinal malabsorption, unspecified

Z98.0 Intestinal bypass and anastomosis status

Z98.84 Bariatric surgery status

**Obesity**

E66.0 Obesity due to excess calories

E66.01 Morbid (severe) obesity due to excess calories

E66.09 Other obesity due to excess calories

E66.1 Drug-induced obesity

E66.8 Other obesity

E66.9 Obesity, unspecified

Z68.3 Body mass index [BMI] 30-39, adult

Z68.30 Body mass index [BMI]30.0-30.9, adult

Z68.31 Body mass index [BMI] 31.0-31.9, adult

Z68.32 Body mass index [BMI] 32.0-32.9, adult

Z68.33 Body mass index [BMI] 33.0-33.9, adult

Z68.34 Body mass index [BMI] 34.0-34.9, adult

Z68.35 Body mass index [BMI] 35.0-35.9, adult

Z68.36 Body mass index [BMI] 36.0-36.9, adult

Z68.37 Body mass index [BMI] 37.0-37.9, adult

Z68.38 Body mass index [BMI] 38.0-38.9, adult

Z68.39 Body mass index [BMI] 39.0-39.9, adult

Z68.4 Body mass index [BMI] 40 or greater, adult

Z68.41 Body mass index [BMI]40.0-44.9, adult

Z68.42 Body mass index [BMI] 45.0-49.9, adult

Z68.43 Body mass index [BMI] 50.0-59.9, adult

Z68.44 Body mass index [BMI] 60.0-69.9, adult

Z68.45 Body mass index [BMI] 70 or greater, adult
