# Supplementary material for: Evaluation of Genetic Associations with Clinical Phenotypes of Kidney Stone Disease
Source: Eur Urol Open Sci. 2024 Jul 24;67:38–44. doi: 10.1016/j.euros.2024.07.109 (PMC11327546; doi:10.1016/j.euros.2024.07.109)
Supplement: Supplementary Table 3 [file mmc3.docx]

| Supplementary Table 3: Demographic and clinical characteristics of genotyped individuals with kidney stone disease | |
| --- | --- |
|  | **Kidney stone diagnosis (n=5571)** |
| Age at diagnosis (years, mean, SD) | 52.0, 18.0 |
| Male gender | 2858 (51.3%) |
| Race |  |
| White race | 4812, (86.4%) |
| Black | 585 (10.5%) |
| Other/unknown | 174 (3.1%) |
| Ethnicity |  |
| Not Hispanic/Latinx | 5422 97.3% |
| Cardiovascular disease | 1281 23.0% |
| Diabetes Type 2 | 1305 23.4% |
| Gout | 325 5.8% |
| Hypertension | 2906 52.1% |
| Inflammatory bowel disease | 575 10.3% |
| Obesity | 1112 20.0% |
|  | |
