# Supplementary material for: Evaluation of Genetic Associations with Clinical Phenotypes of Kidney Stone Disease
Source: Eur Urol Open Sci. 2024 Jul 24;67:38–44. doi: 10.1016/j.euros.2024.07.109 (PMC11327546; doi:10.1016/j.euros.2024.07.109)
Supplement: Supplementary Table 4 [file mmc4.docx]

**Supplementary Table 4.** Linear mixed effects model for age at first kidney stone diagnosis for SNP rs28544423 with sex, race, and ethnicity as fixed effects, and birth cohort (every 10 years) as a random effect.

|  | **Estimate** | **Std. Error** | **df** | **t value** | **Pr(>\|t\|)** |
| --- | --- | --- | --- | --- | --- |
| (Intercept) | 37.96 | 9.22 | 11.57 | 4.12 | 0.00 |
| snp16_20359633 | -0.29 | 0.32 | 973.02 | -0.90 | 0.37 |
| SexM | -0.42 | 0.38 | 973.11 | -1.09 | 0.28 |
| RaceB | 1.99 | 1.87 | 972.98 | 1.07 | 0.29 |
| RaceW | 2.05 | 1.76 | 972.98 | 1.17 | 0.24 |
| EthnicityHL | 1.00 | 3.25 | 972.98 | 0.31 | 0.76 |
| EthnicityNH | 1.46 | 3.07 | 972.98 | 0.48 | 0.63 |
