# Supplementary material for: Evaluation of Genetic Associations with Clinical Phenotypes of Kidney Stone Disease
Source: Eur Urol Open Sci. 2024 Jul 24;67:38–44. doi: 10.1016/j.euros.2024.07.109 (PMC11327546; doi:10.1016/j.euros.2024.07.109)
Supplement: Supplementary Table 5 [file mmc5.docx]

**Supplementary Table 5.** Cox proportions hazards regression model for up to 60 months for the hazard of 2^nd^ stone-related surgery after the 1^st^ stone-related surgery including the SNP rs28544423 allele status.

|  | **Coef** | **exp.coef.** | **se.coef.** | **z** | **Pr…z..** |
| --- | --- | --- | --- | --- | --- |
| snp16_20359633 | 0.03 | 1.03 | 0.14 | 0.24 | 0.81 |
| SexM | -0.37 | 0.69 | 0.17 | -2.17 | 0.03 |
| RaceB | -1.43 | 0.24 | 1.07 | -1.33 | 0.18 |
| RaceI | -15.28 | 0.00 | 3011.35 | -0.01 | 1.00 |
| RaceU | -0.72 | 0.49 | 1.44 | -0.50 | 0.62 |
| RaceW | -1.09 | 0.34 | 1.01 | -1.08 | 0.28 |
| EthnicityHL | 14.03 | 1236891.96 | 1630.49 | 0.01 | 0.99 |
| EthnicityNH | 14.07 | 1293246.17 | 1630.49 | 0.01 | 0.99 |
